# Exchange rate parities and Taylor rule deviations

**DOI:** 10.1007/s00181-021-02192-3

**Published:** 2022-01-11

**Authors:** Christina Anderl, Guglielmo Maria Caporale

**Affiliations:** 1 London South Bank University, London, UK; 2 Department of Economics and Finance, Brunel University London, London, UB8 3PH UK

**Keywords:** PPP, UIP, Nonlinearities, Taylor rules deviations, Inflation targeting, C32, F31, G15

## Abstract

This paper investigates the PPP and UIP conditions by taking into account possible nonlinearities as well as the role of Taylor rule deviations under alternative monetary policy frameworks. The analysis is conducted using monthly data from January 1993 to December 2020 for five inflation-targeting countries (the UK, Canada, Australia, New Zealand and Sweden) and three non-targeting ones (the USA, the Euro Area and Switzerland). Both a benchmark linear VECM and a nonlinear Threshold VECM are estimated; the latter includes Taylor rule deviations as the threshold variable. The results can be summarized as follows. First, the nonlinear specification provides much stronger evidence for the PPP and UIP conditions, the estimated adjustment speed towards equilibrium being twice as fast. Second, Taylor rule deviations play an important role: the adjustment speed is twice as fast when deviations are small and the credibility of the central bank is higher. Third, inflation targeting tends to generate a higher degree of credibility for the monetary authorities, thereby reducing deviations of the exchange rate from the PPP- and UIP-implied equilibrium.

## Introduction

Two well-known puzzles in international finance arise as a result of the apparent failure of many empirical models to find support for either the PPP (Purchasing Power Parity) or the UIP (Uncovered Interest Rate Parity) relations. Various possible explanations have been offered for these findings including: the low power of standard unit root tests (Murray and Papell, 2005); the presence of nonlinearities (Taylor et al., 2001; Kapetanios et al., 2003; Sarno et al., 2006); the failure to take into account the interaction between goods and asset markets (Johansen and Juselius, 1992; Juselius, 1995); non-tradability of goods (Sarno and Chowdhury, 2003) and real frictions (Ford and Horioka, 2017) in the case of PPP; the existence of a risk premium (Li et al., 2012; Biswas et al., 2020); the occurrence of rational bubbles (Obstfeld, 1987; Canterbery, 2000); or deviations from rationality of market participants (Gregory, 1987; Chinn and Quayyum, 2012) in the case of UIP.

Another interesting issue in this context is the possible role of monetary policy regimes. In particular, a few studies have analysed the impact of Taylor rules on PPP (Kim et al., 2014) or UIP (e.g. Backus et al., 2010) separately. By contrast, the present paper aims to assess jointly the empirical validity of PPP and UIP under different monetary policy setups. Specifically, the analysis is conducted over the period from January 1993 to December 2020 for two sets of countries, the first comprising five economies that have adopted inflation targeting (the UK, Canada, Australia, New Zealand and Sweden), the second including three countries (the USA, the Euro Area and Switzerland) that have chosen instead other monetary policy regimes (see Neumann and Von Hagen, 2002, for a similar sample selection). There are, of course, difficulties in categorising countries as ‘pure’ targeters and non-targeters, and in fact some of the latter included in the present study have at times adopted inflation-targeting monetary policies, whilst some of the former have at times chosen monetary discretion instead of strictly following the targeting rule. Our classification is based on how central banks identify themselves, which seems the most appropriate criterion to adopt for our purposes, namely to assess the importance of central bank credibility (measured by Taylor rule deviations, whether from an official or an implicit policy rule) for the dynamic adjustment towards the PPP and UIP equilibrium.

A linear vector error correction model (VECM) for testing jointly PPP and UIP is estimated in the first instance (Juselius, 1995). Given the evidence on possible nonlinearities in exchange rate behaviour (Taylor et al., 2001), a nonlinear threshold VECM framework is then applied. Under inflation targeting, the credibility of the central bank is particularly important for the successful implementation of monetary policy and may affect the adjustment to long-run PPP and UIP. Deviations from the Taylor rule can be interpreted as an indicator of such credibility (Wilde, 2012); therefore, we use them as the threshold variable between regimes characterised by small and large deviations, respectively, and with different adjustment speeds. Deviations from a Taylor rule may have multiple causes such as changes in the inflation target, in the responsiveness to macroeconomic fundamentals, and in the interest rate target; missing variables that enter the policy function; the effective lower bound on short-term interest rates; measurements error in the output gap, etc. However, many studies suggest that, regardless of the underlying cause, the size of Taylor rule deviations affects the behaviour of exchange rates. For instance, Ince et al. (2016) found that out-of-sample exchange rate predictability is much higher during periods characterised by low Taylor rule deviations. It is also well known that if the central bank is believed to follow a monetary policy rule, large and frequent deviations from the rule can indicate a change in monetary policy and therefore influence expectations of the interest rate path and thus affect exchange rates (Kahn, 2010). For these reasons, it seems appropriate to examine the impact of Taylor rule deviations on the dynamic adjustment towards the PPP- and UIP-implied long-run equilibrium and to interpret them mainly as a measure of central bank credibility. This type of analysis has not been carried out before and therefore represents a novel contribution to the literature on exchange rate determination.

The layout of the paper is as follows: Sect. 2 briefly reviews the relevant literature; Sect. 3 outlines the methodology; Sect. 4 presents the data and discusses the empirical results; Sect. 5 offers some concluding remarks.

## Literature review

Most of the literature on the PPP and UIP puzzles assesses them separately. In the case of PPP, unit root tests of the real exchange rate have produced mixed results, with some studies rejecting the null (Cumby and Obstfeld, 1981; Diebold et al., 1991) and others finding instead evidence of nonstationarity (Hakkio, 1984; MacDonald, 1985). Cointegration tests of the PPP relation have been equally inconclusive (Taylor, 1988, 1992; McNown and Wallace, 1990; Kim, 1990). As for UIP, most studies have reported that the interest rate differential is not an optimal predictor of exchange rate changes (Cumby and Obstfeld, 1981; Taylor, 1987; Mylonidis and Semertzidou, 2010; Londono and Zhou, 2017). A large number of empirical studies have been unable to solve the Meese-Rogoff puzzle, namely the fact that random walk models outperform any other exchange rate models in out-of-sample forecasting (Meese and Rogoff, 1983), although Mark (1995) reports that there is an economically significant predicable component (driven by relative money supply and real income) in long-horizon changes (short-horizon changes being dominated by noise instead).

A possible reason for the lack of strong evidence for PPP and UIP is the need to investigate their joint validity in equilibrium models taking into account the linkages between goods and capital markets. For this purpose, Johansen and Juselius (1992) estimated a five-dimensional multivariate cointegration model for the UK based on the framework developed by Johansen (1991) and concluded that more empirical support can be found for exchange rates parities when allowing for interactions between both types of markets. Since then, several other studies have used a similar approach to test for PPP and UIP. Hunter (1992) dropped the weak exogeneity assumption for oil prices and found two cointegration vectors representing the long-run PPP and UIP relations for the British pound. Camarero and Tamarit (1996) conducted the analysis for Spain and provided some more supportive evidence for PPP and UIP. Juselius (1995) examined the case of the Danish krone, whilst Caporale et al. (2001) also used a FIML framework for the German mark and the Japanese yen, both studies confirming the importance of allowing for cross-market linkages. Jaramillo Franco and Serván Lozano (2012) found two stationary vectors in the case of the Peruvian sol, one representing the joint PPP and UIP equilibrium and the other being an interest rate equation with a risk premium.

More recent studies have provided evidence of nonlinear adjustment to long-run PPP and UIP (Kapetanios et al., 2003; Sarno et al., 2006). For instance, Holmes and Maghrebi (2004) and Kisswani and Nusair (2014) estimated a Logistic Smooth Transition Autoregressive (LSTAR) model for Real Interest Parity (RIP) for selected South-East Asian economies; their results support both PPP and UIP with a nonlinear adjustment. A drawback of the RIP approach to investigating exchange rate parities is that it does not shed light on whether a rejection of the joint null is due to a failure of PPP or UIP or both.

Finally, a few papers have found that Taylor rule deviations, measured as the difference between the actual and the target interest rate, can influence the path of the real exchange rate through their impact on central bank credibility (Wilde, 2012). An exchange rate forecasting model with Taylor rule differentials was found to outperform standard UIP and PPP models, both before and after the global financial crisis of 2007–2008, by Molodtsova and Papell (2009, 2013). If the central bank is believed to follow a monetary policy rule, large and frequent deviations from the rule can indicate a change in monetary policy and therefore influence public expectations of the interest rate path and future inflation rates (Kahn, 2010). Nikolsko-Rzhevskyy et al. (2014) calculated several central bank loss functions and found that the costs of deviations from different types of Taylor rules are large; frequent deviations are seen by agents as a permanent shift in monetary policy and might lead to a loss of central bank credibility and affect the monetary policy transmission mechanism. It is common practice in the Taylor rule literature to use Taylor rule deviations to distinguish between rules-based and discretionary periods. Ince et al. (2016), who investigate out-of-sample exchange rate predictability during periods of low Taylor rule deviations and periods of high Taylor rule deviations, found that out-of-sample exchange rate predictability is much stronger in low deviations eras than in high deviations eras. Given the importance of Taylor rule deviations for exchange rate determination, we assess their role in influencing the adjustment to the UIP and PPP exchange rate equilibrium in the following analysis.

## Empirical framework

### The linear Vector Error Correction Model

As a first step, in order to test jointly for long-run PPP and UIP equilibrium relations and also examine the dynamic adjustment process the following linear Vector Error Correction Model (VECM) is estimated (see Johansen, 1991):

1 $$\begin{array}{*{20}c} {\Delta Y_{t} = \mu + \theta z_{t - 1} + \mathop \sum \limits_{i = 1}^{p - 1} \Phi_{i} \Delta Y_{t - 1} + u_{t} } \\ \end{array}$$

where $Y_{t}$ is a vector including in our case the log of the nominal exchange rate $s_{t}$ (defined as domestic currency units per unit of foreign currency), the interest rate differential $\tilde{i}_{t} = i_{t} - i_{t}^{*}$, which is the difference between the domestic and foreign interest rate, and the inflation differential $\tilde{\pi }_{t} = \pi_{t} - \pi_{t}^{*}$, which is the difference between the domestic and foreign inflation rate; $z_{t - 1}$ is the error correction term representing the long-run equilibrium, $\Delta$ is the difference operator, the ${\Phi }_{i}$ stands for the parameters corresponding to the short-run dynamics, $\theta$ is the adjustment parameter measuring the speed at which the system returns to equilibrium after any deviations from it, and $u_{t}$ stands for the innovations. A model with heterogeneous coefficients could be constructed by including the domestic and foreign interest rate and inflation rate variables separately into Eq. (1), as suggested by Molodtsova and Papell (2009). Note, though, that these authors recommend that the choice between homogeneous and heterogeneous coefficients should be guided by economic theory and previous empirical research. Since it is in fact very common in the literature on PPP and UIP to use inflation and interest rate differentials for the analysis, i.e. to make the homogeneity assumption (see, for instance, Berk and Knot, 2001; Kisswani and Nusair, 2014, we follow the same practice below.

Unit root tests, such as the Dickey Fuller Generalised Least Squares (DF-GLS) test and the Kwiatkowski–Phillips–Schmidt–Shin (KPSS) test, are carried out initially to establish whether the variables are of the same order of integration, and then the existence of long-run linkages is investigated by performing Johansen’s (1991) cointegration tests as appropriate. Model adequacy is assessed by means of various diagnostic tests including the White test for heteroscedasticity, the Breusch-Godfrey Lagrange multiplier (LM) test for serial correlation, the Wald test of regressor endogeneity and the Gregory-Hansen test for cointegration with regime shifts.

### The Threshold Vector Error Correction Model

A natural extension of the linear model is a nonlinear Threshold VECM (TVECM) which includes two regimes identified through a threshold variable and takes the following form (Tsay, 1989):

2 $$\begin{aligned} \Delta Y_{t} & = \left( {\mu_{1} + \theta_{1} z_{t - 1} + \mathop \sum \limits_{i = 1}^{p - 1} \Phi_{1,i} \Delta Y_{t - i} } \right)1\left( {d_{t} \le \gamma } \right) \\ & \;\quad\quad + \left( {\mu_{2} + \theta_{2} z_{t - 1} + \mathop \sum \limits_{i = 1}^{p - 1} \Phi_{2,i} \Delta Y_{t - i} } \right)1\left( {d_{t} > \gamma } \right) + u_{t} \\ \end{aligned}$$

where $d_{t}$ is the threshold variable, $\gamma$ is the threshold value, and the other variables are defined as before. The threshold value is estimated empirically as the one which minimises the residual sum of squares.

In the empirical application below, the threshold variable is calculated as the deviations from the Taylor rule adopted by the monetary authorities. Specifically, for each of the countries under examination we estimate the following three different types of rules by using the Generalised Methods of Moments (GMM) method: the classical Taylor rule, the extended Taylor rule, and a Taylor rule with interest rate smoothing. The classical one can be represented as follows:

3 $$\begin{array}{*{20}c} {i_{t} = \alpha + \beta \left( {E_{t - 1} \pi_{t + 3} - \overline{\pi }} \right) + \delta \left( {E_{t - 1} y_{t + 3} } \right) + u_{t} } \\ \end{array}$$

where $i_{t}$ is the nominal interest rate set by the central bank, $E_{t - 1} \pi_{t + 3}$ is the 3-month ahead central bank’s expectation of the inflation rate, $\overline{\pi }$ is the target inflation rate, $E_{t - 1} y_{t + 3}$ is the 3-month ahead central bank’s expectation of the output gap, and $u_{t}$ is a disturbance term. The output gap is calculated using the Hodrick-Prescott Filter,^1^ which is a standard procedure in this area of the empirical literature (Álvarez and Gómez-Loscos, 2018).

The extended Taylor rule includes the real exchange rate as an additional regressor and can be specified as follows:

4 $$\begin{array}{*{20}c} {i_{t} = \alpha + \beta \left( {E_{t - 1} \pi_{t + 3} - \overline{\pi }} \right) + \delta \left( {E_{t - 1} y_{t + 3} } \right) + \lambda q_{t} + u_{t} } \\ \end{array}$$

where $q_{t}$ is the real effective exchange rate and all other variables are defined as before.

Finally, the Taylor rule with interest rate smoothing takes the following form:

5 $$\begin{array}{*{20}c} {i_{t} = \alpha + \rho i_{t - 1} + \left( {1 - \rho } \right)\left( {\beta \left( {E_{t - 1} \pi_{t + 3} - \overline{\pi }} \right) + \delta \left( {E_{t - 1} y_{t + 3} } \right)} \right) + u_{t} } \\ \end{array}$$

where $i_{t - 1}$ is the one-period lagged interest rate, $\rho$ is the partial adjustment parameter which measures the fraction of the target rate by which the central bank moves the current interest rate in each period, and all other variables are the same as before. Under interest rate smoothing, the central bank changes the interest rate gradually in response to a change in inflation, i.e. $i_{t}$ is moved towards $\overline{i}_{t}$ over time. Forward-looking policymakers are assumed to make their policy decisions based on their one-quarter ahead forecast for the fundamentals. Since expected inflation and output cannot be observed directly, we use the 3 month-ahead average as in most of the existing literature on Taylor rules (see Clarida et al., 1998, 2000).

The GMM approach requires the identification of suitable instruments, which are correlated with the variables on the right-hand side of the Taylor rule equation and uncorrelated with the innovations. For our purposes, we use the first lag of the inflation rate and of the output gap in all cases; in the extended Taylor rule, we also add the first lag of the real exchange rate; finally, in the Taylor rule with interest rate smoothing we include the second lag of the interest rate as well. GMM also requires all variables to be stationary; therefore, we perform both the DF-GLS and KPSS test on the individual series to establish their order of integration.

To select the optimal Taylor rule for each country, we use the J-statistic for overidentifying restrictions which tests the validity of the chosen instruments. A relatively large J-statistic indicates that it is questionable whether the model fulfils the GMM moment conditions (Andrews and Lu, 2001). Next we calculate the deviations from the optimal Taylor rule identified for each country as the difference between the policy rate and the target rate determined by the Taylor rule fundamentals (Wilde, 2012; Nikolsko-Rzhevskyy et al., 2014). We consider Taylor rule deviations individually for each country in our models, but also compute the Taylor rule deviations differential as an alternative threshold variable.

### Tests for threshold-type nonlinearity

Prior to estimating the threshold model, a test for threshold-type nonlinearity has to be carried out. We perform two of the most widely used tests, namely the sup-Wald test and the Bai-Perron test (Balke and Fomby, 1997). The former was proposed by Seo (2008) and can be expressed as follows:

6 $$\begin{array}{*{20}c} {W_{n} = sup_{{r{\Gamma }}} n\left\{ {\frac{{\hat{\sigma }^{2} }}{{\hat{\sigma }^{2} \left( T \right)}} - 1} \right\}} \\ \end{array}$$

where $T$ is the number of time periods, $\hat{\sigma }^{2}$ and $\hat{\sigma }^{2} \left( T \right)$ stand for the residual variance for the model under the null and the alternative hypothesis, respectively, $n$ is the number of observations, and $sup_{{r{\Gamma }}}$ is the supremum. The test searches for a single threshold value over the entire range $\left[ { - \gamma ,\gamma } \right]$ of the threshold variable, where $\gamma = max\left| {z_{t - d} } \right|$ is the threshold value and $z_{t - d}$ is the threshold variable. The threshold search is usually restricted to exclude the bottom and top 15% of the observations in the range. The test is constructed in such a way that the break point corresponds to the minimum sum of squares and the highest Wald statistic. Following Seo (2008), we use block bootstrapping with 1000 replications to deal with the problem that the threshold value is unidentified under the linear null. Note that the sup-Wald test is designed to detect the existence of a nonlinear adjustment process towards the long-run equilibrium which is assumed to be a single linear cointegrating vector.

The Bai-Perron test is based instead on a sequential selection method, which tests for the existence and number of thresholds by minimising the sum of squared residuals at the $m$-partition $\left( {T_{1} , \ldots ,T_{m} } \right)$ of $m$ thresholds, resulting in $m + 1$ regimes. It is an F-Test of the null hypothesis of zero thresholds versus the alternative of one threshold. If the null is rejected, the test can be extended to test sequentially for higher numbers of thresholds. This method allows for the identification of the exact number of thresholds with an external threshold variable (Bai and Perron, 2003). We also carry out some diagnostic tests (specifically the Breusch-Godfrey LM test for serial correlation and the Breusch-Pagan LM test for heteroscedasticity) to check model adequacy in each case.

## Data and empirical results

### Data description

As already mentioned, we investigate five inflation targeting countries (the UK, Canada, Australia, New Zealand and Sweden), and three with other monetary policy arrangements (the USA, the Euro Area and Switzerland) which have often been examined in the literature (Cecchetti and Ehrmann, 1999; Mishkin and Schmidt-Hebbel, 2001; Neumann and Von Hagen, 2002). The series are monthly and span the time period from January 1993 to December 2020.^2^ Inflation is calculated as the annual percentage change in CPI; the data sources for Australia and New Zealand are their respective Reserve Banks; for the other countries the series have been obtained from the OECD Statistics. Interest rates are nominal short-term rates, specifically the monthly averages of daily three-month money market rates; these series have also been taken from the OECD Statistics. The nominal exchange rate series come from the Pacific Exchange Rate Service database. The GDP estimates are used to compute the output gap. Whilst most available GDP series are quarterly, there are monthly ones available on the Federal Reserve Bank of St Louis Economic Database, which are volume estimates of real GDP in national currency.^3^ The real effective exchange rates series are CPI-based measures and are taken from the BIS (Bank for International Settlements) Statistics Warehouse. For the bilateral exchange rate series, pre-1999 data for the Euro Area have been obtained from the PACIFIC Exchange Rate Service; these series are the official ECU basket rates. For the real effective exchange rate series, pre-1999 data have been taken instead from the effective exchange rate indices database of the Bank for International Settlements. Finally, for all other variables, including the inflation rate, the interest rate and output, the source for the pre-1999 data is the OECD Monthly Main Economic Indicator Series Publication. We take the natural logarithm of all variables prior to including them in the empirical models.

### Unit root and cointegration tests

As a first step, we perform the DF-GLS and KPSS unit root tests on the nominal exchange rate, interest rate differential and inflation differential series. The results are reported in Table 1a and 1b and indicate that all series are integrated of the same order $I(1)$.

Therefore we proceed to test for cointegration between the series. The results of the Johansen cointegration trace and eigenvalue tests are reported in Table 2 and show that in each case there exists a single cointegration relation which can be interpreted as being consistent with PPP and UIP simultaneously.

**Table 1:** Unit root test results for individual series

(a)				
	Level series		Differenced series	
	DF-GLS	KPSS	DF-GLS	KPSS
	Nominal exchange rates			
GBPCAD	− 1.535	2.95***	− 4.546***	0.0982
GBPAUD	− 1.913	3.59***	− 4.121***	0.0912
GBPNZD	− 2.166	4.01***	− 4.302***	0.083
GBPSEK	− 1.996	3.5***	− 3.966***	0.0752
CADAUD	− 2.547	2.26***	− 9.382***	0.0554
CADNZD	− 2.115	2.39***	− 9.050***	0.0986
CADSEK	− 2.093	0.948***	− 6.271***	0.0349
AUDNZD	− 2.020	2.24***	− 3.649***	0.0576
AUDSEK	− 2.840	1.81***	− 4.931***	0.0273
NZDSEK	− 2.146	2.26***	− 5.684***	0.0428
USDEUR	− 2.084	3.05***	− 9.568***	0.101
USDCHF	− 2.244	2.89***	− 9.643***	0.0725
EURCHF	− 1.788	4.62***	− 5.670***	0.0995

(b)								
	Level series		Differenced series		Level series		Differenced series	
	DF-GLS	KPSS	DF-GLS	KPSS	DF-GLS	KPSS	DF-GLS	KPSS
	Interest Rate Differentials				Inflation Differentials			
UK-Canada	− 2.432	3.57***	− 4.480***	0.0663	− 2.364	1.21***	− 4.101***	0.0102
UK-Australia	− 1.935	3.07***	− 4.249***	0.104	− 2.031	1.42***	− 4.349***	0.0326
UK-New Zealand	− 1.446	2.56***	− 9.103***	0.0955	− 2.392	1.39***	− 7.074***	0.0263
UK-Sweden	− 2.586	1.94***	− 4.021***	0.0948	− 2.523	1.04***	− 6.496***	0.016
Canada-Australia	− 1.492	4.68***	− 6.940***	0.0709	− 2.364	0.743***	− 4.724***	0.0105
Canada-New Zealand	− 2.118	3.75***	− 8.238***	0.0467	− 2.674	0.815***	− 4.407***	0.0116
Canada-Sweden	− 2.041	2.35***	− 8.851***	0.0966	− 1.778	1.24***	− 4.610***	0.0074
Australia-New Zealand	− 2.627	1.75***	− 6.884***	0.0717	− 2.625	0.604***	− 6.133***	0.0211
Australia-Sweden	− 2.455	1.37***	− 7.455***	0.0987	− 1.397	1.53***	− 6.498***	0.019
New Zealand Sweden	− 2.665	0.954***	− 9.155***	0.0992	− 1.728	1.33***	− 7.407***	0.0181
USA-Euro Area	− 1.882	3.91***	− 6.364***	0.0949	− 2.220	1.14***	− 3.582***	0.0286
USA-Switzerland	− 1.945	3.96***	− 4.652***	0.0712	− 2.555	0.508***	− 6.244***	0.0228
Euro Area-Switzerland	− 2.795	1.11***	− 4.935***	0.0162	− 2.151	0.422***	− 4.809***	0.0156

^***^Significant at 1% level

Critical values:

DF-GLS: 1%: − 3.452; 5%: − 2.876; 10%: − 2.570, KPSS: 1%: 0.216; 5%: 0.146; 10%: 0.119

${H}_{0}:$ variable contains a unit root, ${H}_{0}:$ variable is stationary

${H}_{1}:$ variable is stationary, ${H}_{1}:$ variable is not stationary

**Table 2:** Johansen test for cointegration

	Trace Test			Eigenvalue Test		
	Test 1	Test 2	Test 3	Test 1	Test 2	Test 3
UK-Canada	0.0189**	0.3049	0.2905	0.0231**	0.5082	0.2905
UK-Australia	0.0240**	0.0808	0.9260	0.0015***	0.1681	0.1565
UK-New Zealand	0.0380**	0.6693	0.2834	0.0360**	0.5519	0.2834
UK-Sweden	0.0373**	0.3066	0.3054	0.0486**	0.7100	0.3540
Canada-Australia	0.0047***	0.3884	0.8716	0.0025***	0.2333	0.8716
Canada-New Zealand	0.0118**	0.1329	0.1000	0.0333**	0.2232	0.1000
Canada-Sweden	0.0135**	0.1800	0.3150	0.0047***	0.3751	0.8910
Australia-New Zealand	0.0245**	0.2624	0.6578	0.0399**	0.2080	0.6578
Australia-Sweden	0.0220**	0.2546	0.6729	0.0079***	0.2152	0.4430
New Zealand-Sweden	0.0388**	0.2482	0.6560	0.0201**	0.4559	0.6448
USA-Euro Area	0.0465**	0.5256	0.0999	0.0237**	0.6005	0.9109
USA-Switzerland	0.0152**	0.4826	0.2577	0.0088***	0.5425	0.2577
Euro Area-Switzerland	0.0065***	0.4059	0.8809	0.0006***	0.0921	0.6530

$r$ denotes the cointegration rank and number of significant vectors

Trace Test:

Test 1:${H}_{0}: r=0; {H}_{1}: r=1$; 95% Critical value: 42.92

Test 2:${H}_{0}: r\le 1; {H}_{1}: r=2$; 95% Critical value: 25.87

Test 3:${H}_{0}: r\le 2; {H}_{1}: r=3$; 95% Critical value: 12.52

Eigenvalue Test:

Test 1:${H}_{0}: r=0; {H}_{1}: r=1$; 95% Critical value: 25.82

Test 2:${H}_{0}: r\le 1; {H}_{1}: r=2$; 95% Critical value: 19.39

Test 3:${H}_{0}: r\le 2; {H}_{1}: r=3$; 95% Critical value: 12.52

### The linear model

Table 3 reports the adjustment speeds for the linear VECM. Most of the short-run coefficients are not significant and are therefore not reported. As for the adjustment coefficient $\theta$, in some cases it is only significant and negative in the inflation equations, where its estimated value implies that between 3 and 28% of any deviations from the parity equilibrium is corrected within one month. In other cases, the adjustment instead occurs only in the interest rate equation, where between 3 and 6% of any deviation from the equilibrium is corrected within one month. There is no observable difference in the adjustment speed between inflation targeting and non-targeting economies**.**

**Table 3:** Adjustment Speeds in the linear Vector Error Correction Model

	${\Delta s}_{t}$	${\Delta \stackrel{\sim }{\pi }}_{t}$	${\Delta \tilde{i }}_{t}$
GBPCAD	0.00543	0.428***	0.00871
GBPAUD	0.00750	0.147***	0.00930
GBPNZD	0.00312	0.0846***	− 0.00183
GBPSEK	0.00456	0.194***	0.0126
CADAUD	0.00231***	0.0776***	− 0.00338**
CADNZD	0.00414**	0.134***	− 0.00178
CADSEK	0.00212***	0.0440***	− 0.0001
AUDNZD	− 0.0182	− 0.289***	− 0.0676***
AUDSEK	− 0.00470**	− 0.105***	0.00368
NZDSEK	− 0.0001	0.205***	− 0.00328
USDEUR	− 0.00876***	− 0.0297*	0.0137
USDCHF	0.00003	0.129***	− 0.00354
EURCHF	0.00237*	0.110***	0.0224

^*^Significant at 10% level

**Significant at 5% level

***Significant at 1% level. Standard errors in parentheses

We perform a series of diagnostic tests to establish whether the linear models are data congruent. The results are reported in Table 4 and show that they suffer from heteroscedasticity. Furthermore, the Gregory-Hansen test indicates the presence of regime shifts in several cases. Therefore next we estimate a Threshold VECM (TVECM), where the threshold variable is given by deviations from the Taylor rule since these are an important indicator of central bank credibility and could affect the adjustment towards the long-run equilibrium.

**Table 4:** Misspecification tests for the linear models

	Selected Lag	White Test	Breusch-Godfrey LM Test	Wald test	Gregory-Hansen test
GBPCAD	2	0.0000***	0.9665	0.5998	− 4.76
GBPAUD	2	0.0000***	0.2640	0.0000***	− 5.69**
GBPNZD	1	0.0000***	0.1733	0.8550	− 5.71**
GBPSEK	3	0.0000***	0.3223	0.0135**	− 4.87
CADAUD	1	0.0000***	0.0655*	0.0441**	− 5.92**
CADNZD	1	0.0000***	0.4053	0.2634	− 5.77**
CADSEK	1	0.0000***	0.1711	0.9011	− 4.62
AUDNZD	2	0.0000***	0.1328	0.0229**	− 5.95**
AUDSEK	3	0.0000***	0.3530	0.0000***	− 6.03***
NZDSEK	1	0.0000***	0.2004	0.6425	− 5.71**
USDEUR	2	0.0000***	0.5313	0.0004***	− 4.92
USDCHF	2	0.0000***	0.1919	0.3340	− 5.57**
EURCHF	2	0.0000***	0.1306	0.0357**	− 4.95
White Test for Heteroscedasticity:			Breusch-Godfrey LM Test for serial correlation:		
${H}_{0}:homoscedastic\; errors$			${H}_{0}:\,no\; serial\; correlation$		
${H}_{1}:heteroscedastic\; errors$			${H}_{1}:serial\; correlation$		
Wald F-Test for weak exogeneity:			Gregory-Hansen test for cointegration with regime shifts:		
${H}_{0}:no\; endogeneity$			${H}_{0}: no\; cointegration$		
${H}_{1}:weak\; endogenity$			${H}_{1}: cointegration\; with\; regime\; shifts$		
			Critical values: 10%: −5.23; 5%: −5.50; 1%: −5.97		

^*^Significant at 10% level

**Significant at 5% level

***Significant at 1% level. P-values reported for the first three tests. Test statistic reported for the last test

### Taylor rule deviations

Prior to the estimation of the threshold model, we need to obtain a measure of Taylor rule deviations. As already mentioned, the GMM method, which we use to estimate the Taylor rules, requires all variables to be stationary, wherefore we test the individual series for a unit root using the DF-GLS and KPSS tests. The results of these tests are reported in Table 5. As can be seen, the interest rate and real effective exchange rate series are integrated of order $I(1)$, whilst the inflation rate series and the output gap series are integrated of order $I(0)$.

**Table 5:** Unit root test results for individual series entering the Taylor rule

		Level series		Differenced series	
		DF-GLS	KPSS	DF-GLS	KPSS
Interest Rates	UK	− 2.193	2.64***	− 4.678***	0.0911
	Canada	− 2.092	1.0***	− 6.613***	0.0657
	Australia	− 0.880	4.9***	− 4.820***	0.0918
	New Zealand	− 2.049	2.94***	− 5.188***	0.0821
	Sweden	− 2.428	1.55***	− 4.077***	0.0928
	USA	− 1.557	1.79***	− 3.259***	0.0971
	Euro Area	− 2.134	4.09***	− 4.870***	0.0858
	Switzerland	− 2.672	3.41***	− 5.017***	0.0338
Inflation Rates	UK	− 3.560***	1.42***	− 4.834***	0.0558
	Canada	− 4.352***	0.519***	− 5.291***	0.0091
	Australia	− 3.167**	1.65***	− 4.630***	0.0243
	New Zealand	− 3.919***	1.59***	− 8.055***	0.0284
	Sweden	− 3.497***	0.54***	− 6.205***	0.0204
	USA	− 4.159***	0.329***	− 6.339***	0.0201
	Euro Area	− 3.333**	0.865***	− 5.426***	0.0296
	Switzerland	− 3.396**	0.544***	− 6.557***	0.0251
Output Gap	UK	− 6.043***	0.0177	− 17.168***	0.00177
	Canada	− 5.412***	0.0262	− 9.614***	0.0019
	Australia	− 2.899**	0.0192	− 14.327***	0.00247
	New Zealand	− 3.690***	0.253	− 14.276***	0.0111
	Sweden	− 3.131**	0.0131	− 5.109***	0.00209
	USA	− 3.926***	0.0223	− 9.674***	0.00232
	Euro Area	− 4.043***	0.0282	− 25.430***	0.00272
	Switzerland	− 7.682***	0.0223	− 11.560***	0.00162
Real effective exchange rates	UK	− 1.618	4.32***	− 4.991***	0.0758
	Canada	− 1.654	4.47***	− 3.773***	0.017
	Australia	− 1.906	2.76***	− 11.342***	0.0642
	New Zealand	− 2.497	1.25***	− 9.201***	0.0648
	Sweden	− 2.593	1.56***	− 6.511***	0.0482
	USA	− 1.637	3.56***	− 5.051***	0.0989
	Euro Area	− 2.010	2.32***	− 11.045***	0.0991
	Switzerland	− 1.706	4.81***	− 4.887***	0.0715
DF-GLS: 1%: -3.452; 5%: -2.876; 10%: -2.570			KPSS: 1%: 0.216; 5%: 0.146; 10%: 0.119		
${H}_{0}:$ variable contains a unit root			${H}_{0}:$ variable is stationary		
${H}_{1}:$ variable is stationary			${H}_{1}:$ variable is not stationary		

^***^significant at 1% level

Therefore the $I(1)$ series are included in the GMM model in first differences and the $I(2)$ series are included in second differences.^4^ The results of the GMM Taylor rule estimations for the individual countries are reported in Tables 6a and b.

**Table 6:** (a) GMM Results for Individual Taylor Rules in Countries with Alternative Monetary Regimes. (b) GMM Results for Individual Taylor Rules in Inflation Targeting Countries

		$\alpha$	$\beta$	$\delta$	$\lambda$	$\rho$
(a)						
**USA**	Classical	− 2.636***	2.309***	− 6.333		
		(0.694)	(0.419)	(2.326)		
	**Extended**	− 15.801***	1.337***	− 4.166***	2.776***	
		(1.823)	(0.401)	(0.196)	(0.325)	
	Smoothing	− 0.125**	0.0881**	− 3.190		0.980***
		(0.059)	(0.034)	(0.253)		(0.0061)
**Euro Area**	Classical	0.715	0.604	37.804		
		(0.789)	(1.636)	(27.415)		
	**Extended**	2.017	0.615	37.004***	− 5.412***	
		(18.690)	(1.522)	(6.581)	(0.262)	
	Smoothing	0.0006	0.0163	− 0.750		0.988***
		(0.0111)	(0.078)	(4.219)		(0.0413)
**Switzerland**	**Classical**	− 0.342***	1.183***	1.622***		
		(0.086)	(0.112)	(0.288)		
	Extended	24.965***	0.192***	0.003	− 0.287	
		(1.223)	(0.073)	(2.225)	(4.028)	
	Smoothing	− 0.016	0.0193	0.3118		0.985***
		(0.016)	(0.024)	(0.529)		(0.009)

**(b)**						
**UK**	Classical	0.742***	0.509***	− 1.363***		
		(0.182)	(0.169)	(0.293)		
	**Extended**	− 23.767***	1.116***	0.132***	5.062***	
		(0.778)	(0.090)	(0.0161)	(0.164)	
	Smoothing	− 0.009	0.0037	0.0406		0.999***
		(0.013)	(0.0118)	(0.119)		(0.003)
**Canada**	Classical	0.466	0.828**	− 3.231***		
		(0.358)	(0.366)	(0.707)		
	**Extended**	8.340***	0.820**	− 4.071***	− 1.778***	
		(1.756)	(0.368)	(1.758)	(0.377)	
	Smoothing	− 0.0019	0.0083	− 0.130***		0.991***
		(0.022)	(0.0233)	(0.025)		(0.007)
**Australia**	Classical	1.068***	0.481***	0.161***		
		(0.155)	(0.124)	(0.014)		
	**Extended**	2.194***	0.474***	0.137***	− 0.250***	
		(0.704)	(0.125)	(0.007)	(0.038)	
	Smoothing	− 0.014	-0.0194**	0.038***		1.019***
		(0.012)	(0.009)	(0.008)		(0.009)
**New Zealand**	Classical	1.319***	0.369***	16.475		
		(0.067)	(0.067)	(11.351)		
	**Extended**	11.336***	0.298***	27.223**	− 2.159***	
		(1.933)	(0.066)	(12.809)	(0.416)	
	Smoothing	− 0.0131	0.0148	0.0625		0.992***
		(0.0116)	(0.0106)	(0.1664)		(0.004)
**Sweden**	Classical	0.373**	0.712***	− 0.405***		
		(0.184)	(0.163)	(0.109)		
	**Extended**	− 32.366***	0.7881***	0.434***	7.074***	
		(3.137)	(0.139)	(0.127)	(0.668)	
	Smoothing	− 0.0131	0.0148	0.062		0.992***
		(0.0116)	(0.011)	(0.166)		(0.004)

Standard errors in parentheses. Selected Taylor rule models in bold

The instruments are the first lag of the inflation gap and the output gap. These variables are exogenous with respect to the interest rate, and they are generally considered useful for forecasting both inflation and the output gap (Clarida et al., 1998; Taylor and Davradakis, 2006; Caporale et al., 2018). In the extended Taylor rule, the first lag of the real exchange rate serves as an additional instrument, and in the interest rate smoothing Taylor rule, the second lag of the interest rate serves as an additional instrument. We account for heteroscedasticity and autocorrelation by using Newey-West consistent errors. All models are exactly identified. Model selection according to the J-statistic

The optimal Taylor rule is selected by using the J-statistic of overidentifying restrictions. This turns out to be the extended Taylor rule in all cases except Switzerland, for which the classical Taylor rule is instead selected. Our findings are consistent with those of other studies, since the extended Taylor rule, which includes the real exchange rate, should provide a more accurate description of monetary policy than the classical rule in open-economy inflation targeting countries (Svensson, 2000). The only exception is Switzerland, for which, in contrast to other studies (see, for instance, Roth, 2008; Jordan, 2016), we find that the real exchange rate does not matter much in the policy rule. Similar results to ours were reported by Elkhoury (2005), who underlined the decreasing emphasis on the exchange rate compared to other variables in the Swiss policy rule from the 1990s onwards. Most central banks do not publish their Taylor rule projections; these are available only in the case of the US Fed and only at a quarterly frequency, whilst our estimates are monthly; as a result, a direct comparison is not possible; however, it is noticeable that the US Fed reports similar coefficient sizes to ours for the inflation gap over the entire sample range and for the output gap since 2009.^5^

It should be noted that the USA, Switzerland and the Euro Area have in recent years gone through an extended period with interest rates constrained by an effective lower bound. In fact Molodtsova and Papell (2013) compared the out-of-sample exchange rate predictability of Taylor rules taking into account financial conditions indices that summarize information about the future state of the economy with that of conventional Taylor rule models, and found that the former outperform the latter. However, analysing the impact of this new policy framework would require the estimation of the shadow policy rate for which various methods have been proposed, ranging from simple yield curve measures (Black, 1995) to more complex models based on a variety of variables which reflect monetary policy actions (Lombardi and Zhou, 2014); this issue, though, is beyond the scope of the present paper. It should also be acknowledged that monetary policy in the countries under examination had already undergone various other changes since the 1990s and thus it would be interesting to allow for time variation in the parameters of interest. Nevertheless, estimating a constant parameter model is common in the literature computing Taylor rule deviations for various countries, including the USA (Wilde, 2012; Nikolsko-Rzhevskyy et al., 2014), and thus we follow this widespread practice in the present study, whilst future work will also consider time-varying parameter models. Next we calculate the Taylor rule deviations in a similar manner to Wilde (2012) and Nikolsko-Rzhevskyy et al. (2014), namely as the difference between the central bank interest rate and the target interest rate which is determined by the Taylor rule fundamentals.

### Nonlinearity tests

The results of the threshold-type nonlinearity tests (both the sup-Wald and the Bai-Perron tests) are reported in Table 7. In all cases, a single threshold is identified and therefore there exist two regimes characterised respectively by small and large Taylor rule deviations. The estimated coefficients indicate that there is a significant error correction mechanism only in the inflation equations and therefore we focus on the differences in the adjustment speed only in this case. This finding suggests that whilst there is a connection between goods and asset markets, the adjustment occurs only in the former.

**Table 7:** Nonlinearity test and model selection

	Threshold variable	Lag	sup-Wald Test	Bai-Perron threshold test
GBPCAD	UK Taylor rule deviation	3	0.0000***	36.66**
	CA Taylor rule deviation	3	0.0000***	69.80**
GBPAUD	UK Taylor rule deviation	3	0.0000***	27.71**
	AU Taylor rule deviation	3	0.0000***	37.08**
GBPNZD	UK Taylor rule deviation	3	0.0000***	39.93**
	NZ Taylor rule deviation	3	0.0000***	58.77**
GBPSEK	UK Taylor rule deviation	3	0.0000***	47.77**
	SE Taylor rule deviation	3	0.0000***	34.61**
CADAUD	CA Taylor rule deviation	3	0.0000***	39.68**
	AU Taylor rule deviation	3	0.0000***	37.97**
CADNZD	CA Taylor rule deviation	3	0.0000***	44.83**
	NZ Taylor rule deviation	3	0.0000***	37.88**
CADSEK	CA Taylor rule deviation	3	0.0000***	31.99**
	SE Taylor rule deviation	3	0.0000***	32.37**
AUDNZD	AU Taylor rule deviation	3	0.0000***	96.64**
	NZ Taylor rule deviation	3	0.0000***	43.66**
AUDSEK	AU Taylor rule deviation	3	0.0000***	37.03**
	SE Taylor rule deviation	3	0.0000***	51.11**
NZDSEK	NZ Taylor rule deviation	3	0.0000***	41.06**
	SE Taylor rule deviation	3	0.0000***	33.73**
USDEUR	US Taylor rule deviation	3	0.0000***	34.20**
	EU Taylor rule deviation	3	0.0000***	27.19**
USDCHF	US Taylor rule deviation	3	0.0000***	49.81**
	CH Taylor rule deviation	3	0.0000***	61.98**
EURCHF	EU Taylor rule deviation	3	0.0000***	32.76**
	CH Taylor rule deviation	3	0.0000***	29.24**
UK = United Kingdom; CA = Canada; AU = Australia; NZ = New Zealand; SE = Sweden; USA = United States of America; EU = Euro Area; CH = Switzerland				
Sup-Wald test hypothesis:			Bai-Perron 5% Critical Value for Threshold Test: 27.03	
${H}_{0}: linear\; error\; correction$			${H}_{0}: zero\; thresholds$	
${H}_{1}: threshold\; error\; correction$			${H}_{1}: one\; threshold$	

For comparison purposes, we obtain estimates of the adjustment speeds from models including as the threshold variable Taylor rule deviations differentials as well as models incorporating individual Taylor rule deviations instead. To avoid any endogeneity issues the models are specified to include the first lag (rather than the current value) of the Taylor rule deviations and Taylor rule deviations differentials as the threshold variable. Table 8 reports the threshold value for each model along with the adjustment coefficient in the inflation equation for both regimes, where regime one and two correspond, respectively, to small and large Taylor rule deviations. It can be seen that the adjustment speed is twice as fast in the former (when Taylor rule deviations are small) compared to the latter (when Taylor rule deviations are large). For some models, the adjustment only occurs with small Taylor rule deviations, i.e. the error correction coefficient is only significant in regime one. When Taylor rule deviations are small between 6 and 49% of any deviations from the PPP- and UIP-implied equilibrium is corrected within one month; the corresponding percentages for large Taylor rule deviations are 6% and 26%.

**Table 8:** Differences in adjustment speed between regimes in the inflation equation

	Threshold variable ${d}_{t}$	Threshold value $\gamma$	$\theta$ in Regime 1	$\theta$ in Regime 2
GBPCAD	UK Taylor rule deviation	0.300171	− 0.274166***	− 0.135709*
	CA Taylor rule deviation	0.7622549	− 0.243679***	− 0.013683
	Taylor rule differential	− 0.80471131	− 0.038687	− 0.256919***
GBPAUD	UK Taylor rule deviation	0.2879452	− 0.058061**	− 0.237085***
	AU Taylor rule deviation	− 0.41961281	− 0.127265	− 0.076353
	Taylor rule differential	0.84601369	− 0.072450**	− 0.084922
GBPNZD	UK Taylor rule deviation	0.243953	− 0.095953**	− 0.064611
	NZ Taylor rule deviation	0.1810148	− 0.167472***	0.008827
	Taylor rule differential	0.5164786	− 0.041779	− 0.268968***
GBPSEK	UK Taylor rule deviation	− 0.2535326	− 0.270582***	− 0.021266
	SE Taylor rule deviation	− 0.55552321	− 0.416322***	− 0.049830
	Taylor rule differential	− 0.4496821	0.069874	− 0.122436***
CADAUD	CA Taylor rule deviation	0.6146878	− 0.276879***	− 0.219053***
	AU Taylor rule deviation	− 0.01743474	− 0.490511***	− 0.084181**
	Taylor rule differential	0.60670029	− 0.283513***	− 0.049576
CADNZD	CA Taylor rule deviation	0.56581869	− 0.225611***	− 0.036441
	NZ Taylor rule deviation	− 0.3332016	− 0.403656***	− 0.018547
	Taylor rule differential	0.04509599	− 0.221825***	− 0.124470**
CADSEK	CA Taylor rule deviation	0.77194279	− 0.213092***	− 0.171237***
	SE Taylor rule deviation	0.76292289	− 0.170653***	− 0.037323
	Taylor rule differential	− 0.6558203	− 0.363307***	− 0.092615**
AUDNZD	AU Taylor rule deviation	− 0.1130273	− 0.005889	− 0.092306**
	NZ Taylor rule deviation	0.1810148	− 0.133428***	− 0.008973
	Taylor rule differential	0.4352725	− 0.084280**	− 0.070539
AUDSEK	AU Taylor rule deviation	0.04329399	− 0.120272*	− 0.141309***
	SE Taylor rule deviation	− 0.58101951	− 0.350326***	− 0.0665*
	Taylor rule differential	− 0.6289566	− 0.204070	− 0.140641***
NZDSEK	NZ Taylor rule deviation	− 0.398792	− 0.297649***	− 0.068741
	SE Taylor rule deviation	− 0.42736211	− 0.270565***	− 0.071567
	Taylor rule differential	− 0.4730686	− 0.111722	− 0.056862**
USDEUR	US Taylor rule deviation	− 0.16586341	− 0.057533	− 0.078496
	EU Taylor rule deviation	− 0.5393496	− 0.046587	− 0.177176***
	Taylor rule differential	− 0.216241	− 0.179087***	− 0.067315
USDCHF	US Taylor rule deviation	− 0.1569658	− 0.136825***	0.013685
	CH Taylor rule deviation	− 0.28286	− 0.210557***	− 0.029329
	Taylor rule differential	− 0.48040441	− 0.017965	− 0.153485***
EURCHF	EU Taylor rule deviation	− 3.0128771	0.121994	− 0.103428***
	CH Taylor rule deviation	− 0.201426	− 0.298680***	− 0.059835
	Taylor rule differential	− 2.9441171	0.091334	− 0.109353***

Threshold value $\gamma$ with ${d}_{t}$ as the threshold variable

${\varvec{\theta}}$= adjustment coefficient in the inflation equation

UK = United Kingdom; CA = Canada; AU = Australia; NZ = New Zealand; SE = Sweden; USA = United States of America; EU = Euro Area; CH = Switzerland

It would appear that small Taylor rule deviations are seen by agents as pointing to temporary monetary policy discretion, whilst large deviations are perceived as an indication of a permanent shift in monetary policy (Neuenkirch and Tillmann, 2014; Kahn, 2010), which lowers the adjustment speed to PPP and UIP. When using the Taylor rule differentials variable as the threshold variable, the adjustment speed for some exchange rates is higher in regime two, i.e. when there is a divergence in Taylor rule deviations between the two countries. This suggests that the adjustment to the UIP and PPP equilibrium is more strongly influenced by Taylor rule deviations occurring in some countries than in others, and that considering such deviations for each country separately in the models provides a more conclusive picture of their role in influencing the adjustment towards equilibrium.

In inflation targeting countries, the adjustment in the small Taylor rule deviations regime is more than twice as fast as in non-targeting ones—more precisely, between 6 and 49% of any deviations from the PPP- and UIP-implied equilibrium is corrected within one month, the corresponding percentages for non-targeting economies being 10% and 20%. Whilst deviations from the monetary policy rule still matter for the adjustment to the UIP and PPP equilibrium in non-targeting countries, it seems that the central bank commitment to prioritise inflation targeting as the main monetary policy objective generates a significantly faster adjustment, provided that Taylor rule deviations are low. The reason might be that in the inflation targeting regime agents have a reference point against which expectations can be measured. This suggests that monetary authorities are able to achieve greater credibility and reduce deviations from the UIP and PPP equilibrium when they implement policies which adhere closely to the inflation target.

Finally, we check the adequacy of the nonlinear models by testing for serial correlation, heteroscedasticity and parameter stability. The latter is particularly important as our sample period includes the Covid-19 pandemic that could have affected the foreign exchange market (see, e.g. Salisu and Vo, 2020, for some evidence of the effects of the pandemic on stock markets). The results of these tests are reported in Table 9 and confirm the data congruency of the nonlinear models. In particular, Cumulative Sum (CUSUM) tests suggest that the regression parameters are stable over the sample period and thus there is no evidence of an impact of the recent Covid-19 pandemic.

**Table 9:** Diagnostic Tests for the Nonlinear Model

	Threshold variable	Breusch-Godfrey Test for Serial Correlation	Breusch-Pagan Test for Heteroscedasticity	CUSUM Test for Parameter Constancy
GBPCAD	UK Taylor rule deviation	0.4579	0.1355	p-value > 0.05
	CA Taylor rule deviation	0.8607	0.0541	p-value > 0.05
	Taylor rule deviation differential	0.7416	0.5487	p-value > 0.05
GBPAUD	UK Taylor rule deviation	0.9092	0.4631	p-value > 0.05
	AU Taylor rule deviation	0.9256	0.5355	p-value > 0.05
	Taylor rule deviation differential	0.3594	0.5416	p-value > 0.05
GBPNZD	UK Taylor rule deviation	0.1983	0.0002***	p-value > 0.05
	NZ Taylor rule deviation	0.7320	0.7095	p-value > 0.05
	Taylor rule deviation differential	0.1542	0.0943	p-value > 0.05
GBPSEK	UK Taylor rule deviation	0.9354	0.6603	p-value > 0.05
	SE Taylor rule deviation	0.4739	0.3928	p-value > 0.05
	Taylor rule deviation differential	0.4370	0.0197**	p-value > 0.05
CADAUD	CA Taylor rule deviation	0.9317	0.3451	p-value > 0.05
	AU Taylor rule deviation	0.1036	0.9104	p-value > 0.05
	Taylor rule deviation differential	0.9657	0.9295	p-value > 0.05
CADNZD	CA Taylor rule deviation	0.1734	0.2481	p-value > 0.05
	NZ Taylor rule deviation	0.0770	0.1940	p-value > 0.05
	Taylor rule deviation differential	0.1269	0.2565	p-value > 0.05
CADSEK	CA Taylor rule deviation	0.7972	0.3123	p-value > 0.05
	SE Taylor rule deviation	0.9035	0.0066***	p-value > 0.05
	Taylor rule deviation differential	0.1738	0.1667	p-value > 0.05
AUDNZD	AU Taylor rule deviation	0.3385	0.3464	p-value > 0.05
	NZ Taylor rule deviation	0.9579	0.5929	p-value > 0.05
	Taylor rule deviation differential	0.9959	0.0569	p-value > 0.05
AUDSEK	AU Taylor rule deviation	0.7188	0.1525	p-value > 0.05
	SE Taylor rule deviation	0.8318	0.1339	p-value > 0.05
	Taylor rule deviation differential	0.0569	0.2613	p-value > 0.05
NZDSEK	NZ Taylor rule deviation	0.6249	0.3669	p-value > 0.05
	SE Taylor rule deviation	0.1419	0.2664	p-value > 0.05
	Taylor rule deviation differential	0.7507	0.1648	p-value > 0.05
USDEUR	US Taylor rule deviation	0.6617	0.0120**	p-value > 0.05
	EU Taylor rule deviation	0.5197	0.4233	p-value > 0.05
	Taylor rule deviation differential	0.0555	0.8759	p-value > 0.05
USDCHF	US Taylor rule deviation	0.1014	0.5778	p-value > 0.05
	CH Taylor rule deviation	0.4944	0.9915	p-value > 0.05
	Taylor rule deviation differential	0.5170	0.5634	p-value > 0.05
EURCHF	EU Taylor rule deviation	0.9083	0.0917	p-value > 0.05
	CH Taylor rule deviation	0.4420	0.4361	p-value > 0.05
	Taylor rule deviation differential	0.0934	0.2358	p-value > 0.05
Breusch-Godfrey LM Test for serial correlation:			Breusch-Pagan LM Test for heteroscedasticity:	
${H}_{0}: no\; serial\; correlation$			${H}_{0}:homoscedasticity$	
${H}_{1}: serial\; correlation$			${H}_{1}:heteroscedasticity$	
CUSUM Test for parameter constancy:				
$${H}_{0}: parameter\; constancy$$				
$${H}_{1}: no\; parameter\; constancy$$				

UK = United Kingdom; CA = Canada; AU = Australia; NZ = New Zealand; SE = Sweden; USA = United States of America; EU = Euro Area; CH = Switzerland

## Conclusions

The aim of this paper is to provide new evidence on the empirical validity of PPP and UIP by taking into account possible nonlinearities and also investigating the role of Taylor rule deviations under alternative monetary policy frameworks. The analysis is conducted using monthly data from January 1993 to December 2020 for five countries that have adopted inflation targeting (the UK, Canada, Australia, New Zealand and Sweden) and also three economies with other monetary regimes (the USA, the Euro Area and Switzerland). Both a benchmark linear VECM and a nonlinear Threshold VECM are estimated; the latter includes Taylor rule deviations as the threshold variable.

The results can be summarised as follows. First, taking into account nonlinearities provides much stronger evidence for the PPP and UIP conditions. In particular, the dynamic adjustment towards equilibrium, which only occurs in the inflation equations, is more than twice as fast compared to the linear case and the joint goods and asset market equilibrium is reinstated through an adjustment taking place in the goods market only. Second, Taylor rule deviations play an important role: the adjustment speed is twice as fast when they are small and are perceived as temporary departures from the monetary policy rule, large deviations being interpreted instead as an indication of permanent shifts in monetary policy. This finding is consistent with those of previous studies (Neuenkirch and Tillmann, 2014; Kahn, 2010) and implies that the credibility of the central bank has an impact on the exchange rate path. Third, the evidence is more supportive of the PPP and UIP parities in inflation targeting countries, where the speed of adjustment towards the long-run equilibrium is twice as fast compared to non-targeting economies. This suggests that, although other differences between these two groups of countries might influence convergence to long-run equilibrium values, the inflation targeting framework tends to generate a higher degree of credibility for monetary authorities, thereby reducing deviations of the exchange rate from the PPP- and UIP-implied equilibrium.

## Footnotes

1 Please note that, although the Hodrick-Prescott Filter is a very commonly used measure of the output gap, it has some limitations; in particular, it has been found to introduce spurious dynamics that are not based on the underlying data generating process in the detrending process (Hamilton, 2018).

2 The official dates when inflation targeting was adopted in each country are as follows: UK—October 1992; Canada—February 1991; Australia—January 1993; New Zealand—December 1989; Sweden—January 1993. Therefore, a sample starting in January 1993 captures most of the inflation targeting period in these countries.

3 Quarterly, but not monthly, output gap estimates are available from central banks, and therefore we need to calculate the latter. The Hodrick-Prescott Filter is applied to the real GDP series in logs. The multiplier λ, which allows to adjust the sensitivity of the trend component to short-term variations, is set to 129,600, which is appropriate for monthly data.

4 We estimate the three Taylor rules according to Eqs. (3), (4) and (5), but, instead of including the integrated in their levels, we now include them in their differences.

5 Many other studies have estimated output gap coefficients of around 0.5 using quarterly data, although coefficients greater than -1 (in absolute value) were found by Petersen (2007) and by Castro (2011). The official US Taylor rule projections by the Federal Reserve Bank of Atlanta also contain output gap coefficients which at times range between -1 and -17 (Federal Reserve Bank of Atlanta, 2021).
